# Supplementary material for: Purcell enhancement of directional edge photocurrent in a van der Waals self-cavity
Source: Nat Commun. 2026 Apr 28;17:3865. doi: 10.1038/s41467-026-72260-8 (PMC13125300; doi:10.1038/s41467-026-72260-8)
Supplement: Supplementary file 1 — Supplementary Information [file 41467_2026_72260_MOESM1_ESM.pdf]

Supplementary information: Purcell enhancement of directional edge photocurrent in a van der Waals self-cavity

Xinyu Li[1,2], Jesse Hagelstein[1,2], Gunda Kipp[1,2], Felix Sturm[1,2,3], Kateryna Kusyak[1,2,3], Yunfei Huang[3], Benedikt Schulte[1,2,3], Alexander M. Potts[1,2,3], Jonathan Stensberg[3,4], Victoria Quirós-Cordero[5], Chiara Trovatello[5,6], Zhi Hao Peng[4], Chaowei Hu[7], Jonathan M. DeStefano[7], Michael Fechner[1,2], Takashi Taniguchi[8], Kenji Watanabe[9], P. James Schuck[5], Xiaodong Xu[7], Jiun-Haw Chu[7], Xiaoyang Zhu[4], Angel Rubio[1,2,10], Marios H. Michael[1,2], Matthew W. Day[1,2,3], Hope M. Bretscher[1,2,3*], James W. McIver[1,2,3*]

[1]Max Planck Institute for the Structure and Dynamics of Matter, Hamburg, Germany.
[2]Center for Free-Electron Laser Science, Hamburg, Germany.
[3]Department of Physics, Columbia University, New York, NY, USA.
[4]Department of Chemistry, Columbia University, New York, NY, USA.
[5]Department of Mechanical Engineering, Columbia University, New York, NY, USA.
[6]Physics Department, Politecnico di Milano, Milan,IT.
[7]Department of Physics, University of Washington, Seattle, WA, USA.
[8]Research Center for Materials Nanoarchitectonics, National Institute for Materials Science, Tsukuba, Japan.
[9]Research Center for Electronic and Optical Materials, National Institute for Materials Science, Tsukuba, Japan.
[10]Initiative for Computational Catalysis, Simons Foundation Flatiron Institute, New York, USA.

*Corresponding author(s). E-mail(s): hope.bretscher@mpsd.mpg.de; jm5382@columbia.edu;

Supplementary Note 1: Crystal symmetry and its impacts on the experimental measurements

The crystal structure of T_d-WTe_2 is shown in Supplementary Figure 1.

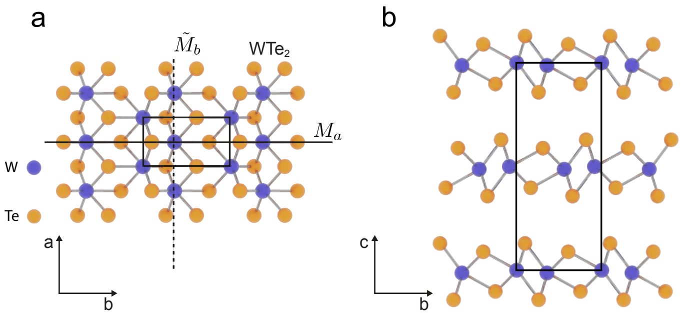

Supplementary Figure 1: **T_d-WTe_2 crystal structure.** Side and top view of T_d-WTe_2 crystal. The black box indicates a crystal unit cell.

In the T_d phase of WTe_2 (space group $Pmn2_1$), two distinct in-plane mirror symmetries are relevant for analysing photocurrent responses:

1. The mirror plane M_a parallel to the a axis, present within each individual crystal layer.

2. The glide mirror plane $\tilde{M}_b$ parallel to the b axis, arising from the multilayer stacking arrangement.

Depending on the specific microscopic mechanism of photocurrent generation, the presence or absence of a given mirror symmetry imposes different constraints on the allowed photocurrent components.

In the main text, we discuss two possible mechanisms that can generate directional edge currents:

(1) photogalvanic currents (PGE), and

(2) photocurrents arising from the anisotropic photothermal effect (APTE).

Here, we analyse the symmetry constraints for each mechanism individually, and then combine them into a unified rule applicable to both.

Photogalvanic current:

PGE can be expressed as [1]:

$$j_\alpha = \sigma_{\alpha\beta\gamma} E_\beta(\omega) E_\gamma(-\omega), \tag{1}$$

where j_α is the DC photocurrent along direction α, $\sigma_{\alpha\beta\gamma}$ is the second-order photoconductivity tensor determined by the crystal's symmetry, and E_β are components of the driving electric field at frequency ω. Crystal symmetries impose strict constraints on $\sigma_{\alpha\beta\gamma}$.

We consider a system that is invariant under the mirror operation M_{xz} (mirror plane in the xz plane), which acts on real-space coordinates as

$$(x, y) \xrightarrow{M_{xz}} (x, -y). \tag{2}$$

Here, we restrict the notation to the in-plane coordinates x and y without loss of generality.

Under this reflection, the y component of any polar vector changes sign, while the x and z components remain unchanged. For the current density $\mathbf{j}$, this transformation reads

$$\begin{aligned} (M_{xz}\mathbf{j})_x (x, y) &= +\, j_x(x, -y), \\ (M_{xz}\mathbf{j})_y (x, y) &= -\, j_y(x, -y). \end{aligned} \tag{3}$$

Equivalently, this can be written as

$$j_x \xrightarrow{M_{xz}} j_x, \qquad j_y \xrightarrow{M_{xz}} -j_y. \tag{4}$$

The same transformation applies to the electric field, such that

$$E_y \xrightarrow{M_{xz}} -E_y. \tag{5}$$

Because the mirror operation leaves the physical system invariant, the photocurrent must transform consistently under M_{xz}. While the left-hand side of Eq. 1 transforms as $j_y \to -j_y$, the right-hand side remains unchanged, since $(E_y)^2 \to (-E_y)^2$. This condition can only be satisfied for all spatial positions (x, y) if $\sigma_{y\beta\gamma} = 0$, which implies

$$\boxed{j_y = 0 \qquad (\text{with } M_{xz}).} \tag{6}$$

Anisotropic photothermal effect (APTE):

We now apply the same mirror-symmetry analysis to the anisotropic photothermal effect. Assuming that the crystallographic a axis is aligned with the x direction, the photocurrent components can be written as [2]:

$$\begin{aligned} J_x(\mathbf{r}) &= -\sigma_a \left[\partial_x \Phi(\mathbf{r}) + S_a\, \partial_x T(\mathbf{r})\right], \\ J_y(\mathbf{r}) &= -\sigma_b \left[\partial_y \Phi(\mathbf{r}) + S_b\, \partial_y T(\mathbf{r})\right], \end{aligned} \tag{7}$$

where $\sigma_{a/b}$ and $S_{a/b}$ denote the electrical conductivities and Seebeck coefficients along the a and b axes, respectively.

Here, $\Phi(\mathbf{r})$ denotes the electrochemical potential induced by laser excitation, obtained from the continuity equation

$$\nabla \cdot \mathbf{J} = 0, \tag{8}$$

with the boundary conditions $\Phi \to 0$ far from the excitation region and $\mathbf{J} \cdot \mathbf{n} = 0$ at the sample edges ($\mathbf{n}$: local outward normal).

The total photocurrent is the sum of a diffusion term,

$$\mathbf{J}_{\mathrm{d}} = -\boldsymbol{\sigma}\,\nabla\Phi, \tag{9}$$

and a photothermal term,

$$\mathbf{J}_{\mathrm{ph}} = -\boldsymbol{\sigma}\,\mathbf{S}\,\nabla T, \tag{10}$$

which arises from laser-induced temperature gradients.

For an isotropic Gaussian temperature profile $T(\mathbf{r}) \propto e^{-r^2}$, a finite anisotropy ($S_a \neq S_b$) generates circulating current patterns in the bulk, with $|\mathbf{J}| \propto |S_a - S_b|$; examples simulated under the same formalism are shown in Supplementary Figure 2.

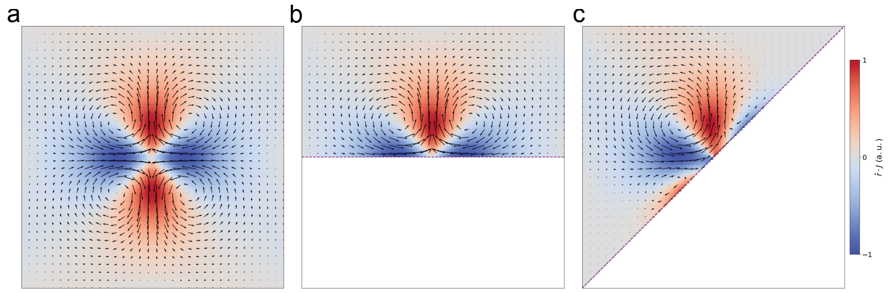

Supplementary Figure 2: **Simulated photocurrent distributions under different excitation conditions**. Adapted from Ref. [2], with simulations performed using the same theoretical formalism and boundary conditions. Simulated current distributions assuming a Gaussian temperature profile $T(\mathbf{r}) \propto e^{-r^2}$ following laser excitation, with $S_a \neq S_b$ and $\sigma_a = \sigma_b$. Different excitation conditions are considered, including bulk excitation (a) and excitation at edges oriented along the $\langle 100\rangle$ (a axis) (b) and $\langle 110\rangle$ (c) directions. The radial flow component $\mathbf{J}\cdot\hat{\mathbf{r}}$ is shown as a false-colour map. The electrochemical potential Φ is obtained numerically using Python.

We again consider a mirror plane M_{xz}. Solving Eq. (8) using Eq. (7) gives

$$\sigma_a\,\partial_x^2\Phi + \sigma_b\,\partial_y^2\Phi = -\,\sigma_a S_a\,\partial_x^2 T - \sigma_b S_b\,\partial_y^2 T. \tag{11}$$

If the temperature profile is invariant under M_{xz}, the solution $\Phi(x, y)$ also obeys the same mirror symmetry:

$$T(x,-y) = T(x,y), \qquad \Phi(x,-y) = \Phi(x,y). \tag{12}$$

Differentiation then yields

$$\begin{aligned} \partial_x\Phi(x,-y) &= \partial_x\Phi(x,y), & \partial_x T(x,-y) &= \partial_x T(x,y),\\ \partial_y\Phi(x,-y) &= -\,\partial_y\Phi(x,y), & \partial_y T(x,-y) &= -\,\partial_y T(x,y). \end{aligned} \tag{13}$$

Substituting into Eq. (7) gives the mirror-symmetry relations:

$$\boxed{\begin{aligned} J_x(x,-y) &= J_x(x,y), \\ J_y(x,-y) &= -\,J_y(x,y). \end{aligned}} \tag{14}$$

Equation (14) shows that the presence of the M_{xz} mirror plane imposes a clear constraint on the APTE photocurrent distribution: while it does not locally forbid a given current component, it enforces symmetry of the spatial pattern across the mirror plane. Consequently, the net photocurrent measured by integrating local currents along a detection direction will reflect these symmetry restrictions.

Summary of mirror-plane symmetry constraints and application to WTe_2 crystal

Combining Eq. (6) (PGE) and Eq. (14) (APTE), we arrive at a general rule applicable to both mechanisms: *for a system invariant under a given mirror plane, the net photocurrent—defined as the spatial integral of the local current density—can only have components lying within that mirror plane.*

T_d-phase WTe_2 possesses two in-plane mirror symmetries, M_a and $\tilde{M}_b$. Whether these are preserved in a given measurement geometry determines the allowed photocurrent components. The main symmetry scenarios are summarised in Supplementary Table 1:

1. In the bulk region, the presence of both mirror planes forbids any net photocurrent.
2. For edges parallel to the crystal axes, the allowed net photocurrent is along the mirror-symmetric direction, i.e., perpendicular to the edge.
3. For edges misaligned with the crystal axes, no mirror-plane constraints apply. The allowed net photocurrent can have both perpendicular and parallel components with respect to the edge.

Supplementary Table 1: Symmetry constraints on photocurrent generation in T_d-phase WTe_2.

Excitation position	Preserved mirror symmetry	Allowed net photocurrent
Bulk	M_a and $\tilde{M}_b$	Forbidden
Edge ∥ crystal axis	M_a or $\tilde{M}_b$ perpendicular to edge	Perpendicular to edge only
Generic edge	No mirror plane preserved	Parallel and perpendicular to edge

Supplementary Note 2: Device information

Micrographs and heterostructure details of all devices measured in this manuscript are shown in Supplementary Figure 3.

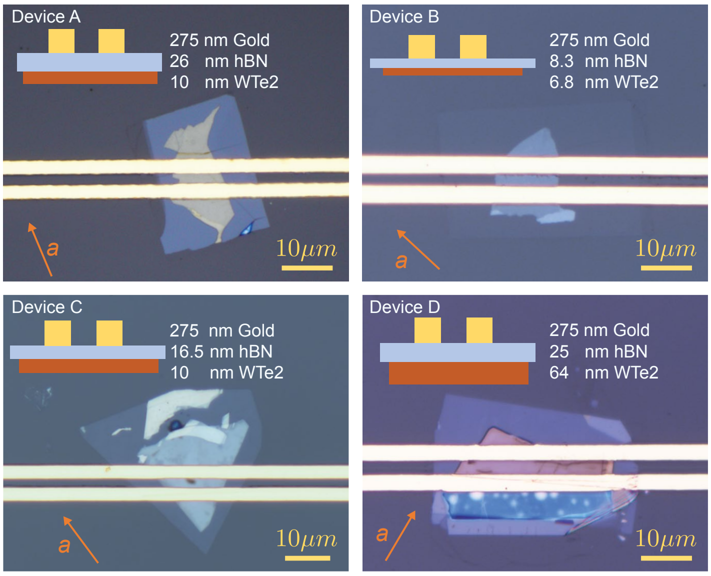

Supplementary Figure 3: **Heterostructure details for Device A to D.** Micrograph of WTe_2 for each Device. Inset shows the layer thickness of the gold co-planar striplines, hBN, and WTe_2 layers. Orange arrows indicate the direction of the a-axis as determined by second harmonic generation (SHG) measurements.

The thicknesses shown in Supplementary Figure 3 correspond to the individual flakes measured by AFM. For clarity, we summarise the parameters in the table below:

Supplementary Table 2: Thickness parameters for Devices A–D. The metal strip consists of 10 nm Ti and 275 nm Au, deposited under identical evaporation conditions, and the amorphous silicon (a-Si) photoconductive switch has a constant thickness of 165 nm.

**Device**	**hBN thickness (nm)**	**WTe_2 thickness (nm)**	**Additional layers**
A	26.0	10.0	10 nm Ti + 275 nm Au & 165 nm a-Si
B	8.3	6.8	10 nm Ti + 275 nm Au & 165 nm a-Si
C	16.5	10.0	10 nm Ti + 275 nm Au & 165 nm a-Si
D	25.0	64.0	10 nm Ti + 275 nm Au & 165 nm a-Si

In all devices, the metal strip thickness is fixed at 10 nm Ti and 275 nm Au (identical deposition parameters), and the a-Si layer thickness is consistently 165 nm. This value is chosen to approximately match the penetration depth of a-Si at the 515 nm laser wavelength used in our experiments.

The crystal axis is determined by second harmonic generation (SHG) measurements, as the SHG signal is known to be maximized along the b crystal axis [3]. For each device, SHG measurements were performed multiple times at different points to enhance accuracy. The final result used for analysis is the statistical average and standard deviation of these measurements. Supplementary Figure 4 presents an example of the measurement trace for each device.

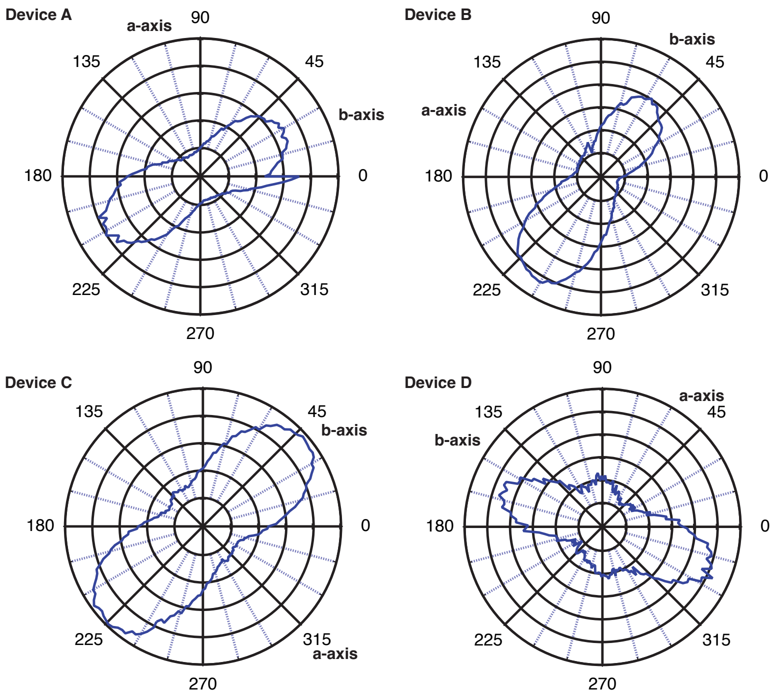

Supplementary Figure 4: **Second harmonic generation for each device.** Examples of second harmonic generation results for each device. 0 degrees corresponds to the angle parallel to the co-planar stripline as shown in Supplementary Figure 3.

Supplementary Note 3: Calculation of emission amplitude, fluence and Fourier transform frequency

In this section, we describe how the peak amplitude and linewidth were extracted as a function of fluence, shown in Fig.3 (main text).

First, the fluence of the pump laser, denoted as $F_{\max}$, was determined from the laser power P using the formula given in [4]:

$$F_{\max}(P) = \frac{P}{f_{\text{ref}}} \cdot \frac{1}{2\pi\sigma_x\sigma_y} \tag{15}$$

Here, $f_{\mathrm{ref}} = 200$ kHz is the laser repetition frequency, which is further halved for the sample excitation beam as this beam is chopped by a 50 % duty cycle optical chopper wheel.

The term $\frac{1}{2\pi\sigma_x\sigma_y}$ represents the spatial integral. The Gaussian widths of the pump beam at the sample plane, σ_x and σ_y, are determined by fitting a 2D Gaussian function to the pump spot, and thus are different for each dataset due to changes in alignment and imaging focus; details for each case are listed in Supplementary Table 3.

Supplementary Table 3: Spot sizes of the pump beam for different devices.

**Device**	**Excitation Position**	σ_x (µm)	σ_y (µm)
A	In between co-planar stripline	2.678±0.091	2.317±0.079
	Outside co-planar stripline	5.22±0.07	5.22±0.06
B	Large beam covering device	12.48±0.06	12.37±0.07
C	In between and outside co-planar stripline	4.77±0.12	4.27±0.11
D	In between co-planar stripline	2.156±0.014	2.074±0.014

The emission signal is determined from the current measured across the photoconductive switch. This current is amplified by a transimpedance amplifier (TIA) and measured using a lock-in amplifier. The bias voltage at the switch can be determined by measuring a calibration curve corresponding to measuring a current as a function of known bias. The bias voltage from a THz emission measurement V_{bias} can then be obtained from the lock-in reading $x_{\text{Lock-in}}$ using the slope of the calibration curve (k_0):

$$V_{\mathrm{bias}} = k_0 \cdot x_{\text{Lock-in}} \tag{16}$$

The emission field E_{emission} is then calculated by dividing by the gap d between the co-planar stripline at the position of the photoconductive switch:

$$E_{\mathrm{emission}} = \frac{k_0}{d} x_{\text{Lock-in}} \tag{17}$$

The width between the metal traces at the photoconductive switch for each device are as given: Device A: $d = 2 \pm 0.1$ µm; Device B: $d = 2.5 \pm 0.1$ µm; Device C: $d = 2.3 \pm 0.1$ µm; Device D: $d = 2.5 \pm 0.1$ µm.

Using the calibration curve and spot size measured for each dataset, we calculated the signal amplitude and excitation laser fluence, and their corresponding error.

The uncertainty for the finite frequency peak was calculated using a different method. For example, for Device A and B in Fig. 3 (main text), we fit the peak in each device with a Lorentzian resonance. An example is given in Supplementary Figure 5 at the highest fluence for each device. Using this fitting, we extracted the value and error of each FWHM. For Device A, we utilized 10 separate traces for 10 measurements. We then extracted the amplitude and peak frequency of each trace to obtain the average and error of these two values. For Device B, individual traces were not

acquired- so the error of a single fit was used to obtain the error for the amplitude and resonance frequency position.

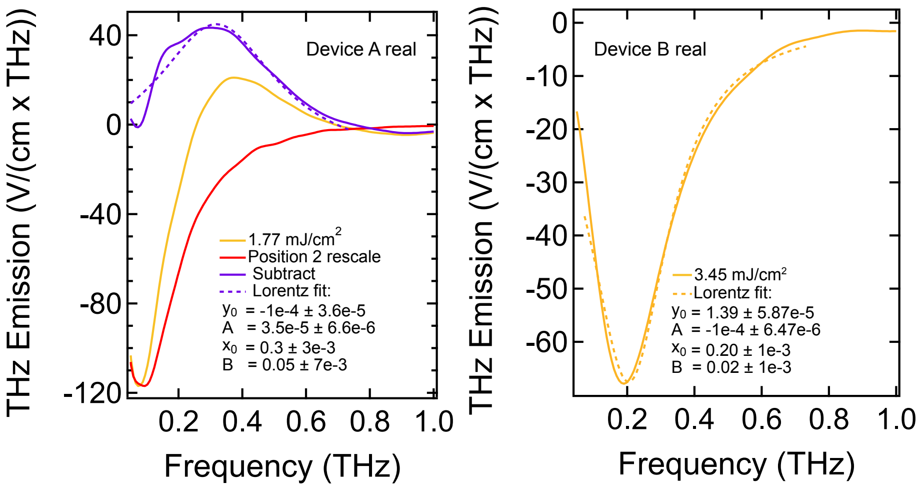

Supplementary Figure 5: Linewidth fitting for Device A and B. For Device A, we utilized the directional photocurrent response from Fig. 2 (main text) to eliminate the DC component from Fig. 3 (main text), and fit the remaining part using a Lorentzian lineshape $y = y_0 + \frac{A}{(x-x_0)^2+B}$ to extract a resonance frequency position, amplitude, and FWHM at each fluence. For Device B, the finite frequency peak dominates the signal, so this was directly fit using a Lorentzian lineshape.

Example of extracting undamped frequency and standard deviation

As an example for Device A, in Fig. 3**c** (main text) lower panel, we show the extracted frequency of the peak maximum at four different fluences ($f_1 = \{0.409, 0.433, 0.384, 0.378\}$ THz, at fluences of $\{0.708,\ 1.06,\ 1.42,\ 1.77\}$ mJ/cm^2, with standard deviation $\sigma_{f_1,static} = \{0.051,\ 0.032,\ 0.025,\ 0.012\}$ THz). For Device A, we have 10 time-domain traces, so f_1 and its standard deviation were determined by obtaining the peak maximum of each trace and calculating the mean and variance. For Device B-D, f_1 was obtained by determining the peak maximum, and the standard deviation is determined by the difference between the max peak position the value found through a Lorentzian fit.

The window used to calculate the Fourier transform, $\sim$ 10 ps, determines the frequency domain resolution. This adds a systematic error of $\sigma_{f_1,\mathrm{sys}} = 0.05/2$ THz, such that the total standard deviation is $\sigma_{f_1} = \sqrt{\sigma^2_{f_1,\mathrm{static}} + \sigma^2_{f_1,\mathrm{sys}}} = \{0.057,\ 0.041,\ 0.036,\ 0.028\}$ THz.

The results of the Lorentzian peak fitting is used to calculate the quality factor, by $Q = f_1/(2\sqrt{B_{\text{Lor}}}) = \{2.3, 1.0, 0.84, 0.82\}$ with $\sigma_Q = \{0.38, 0.14, 0.076, 0.066\}$.

At each fluence, the undamped frequency was calculated using the equation, $f_0 = f_1/\sqrt{1 - 1/(2Q)^2}$. For Device A, the values of f_0 were found to be: {0.420, 0.501, 0.479, 0.478} THz, with corresponding standard deviation σ_{f_0} ={0.059, 0.053, 0.051, 0.042} THz. The four values for f_0 (each under different laser fluence) were used to calculate a weighted mean and standard deviation defined by:

$$\bar{f}_0 = \frac{\sum_{i=1}^{n} \left(\frac{f_{0,i}}{\sigma_i^2}\right)}{\sum_{i=1}^{n} \left(\frac{1}{\sigma_i^2}\right)} \tag{18}$$

$$\sigma_{f_0} = \sqrt{\frac{1}{\sum_{i=1}^{n} \left(\frac{1}{\sigma_i^2}\right)}} \tag{19}$$

to obtain the final result of $f_0 = 0.473 \pm 0.025$ THz.

Supplementary Note 4: Extended data for excitation in between co-planar stripline

The spectral information for the time-domain data shown in Fig. 2**b** (main text) is shown in Supplementary Figure 6.

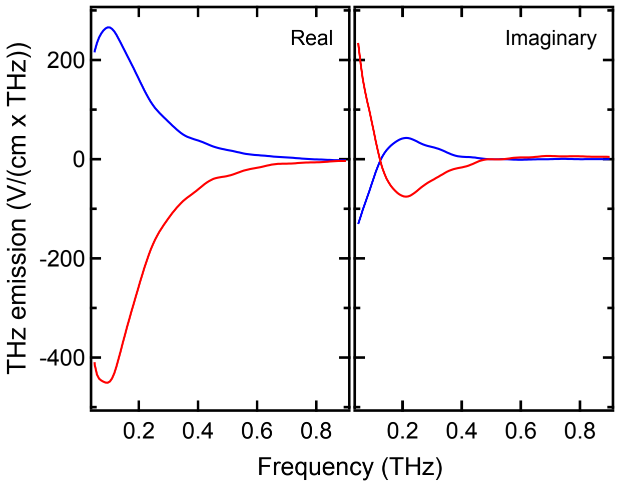

Supplementary Figure 6: Real and imaginary emitted signal as a function of frequency from Device A excited between the stripline traces at the left and right edge of the flake.

The fluence-dependent time domain trace (a) and corresponding frequency domain (b) is shown in Supplementary Figure 7. The excitation fluence did not lead to any differences in frequency dependent behavior.

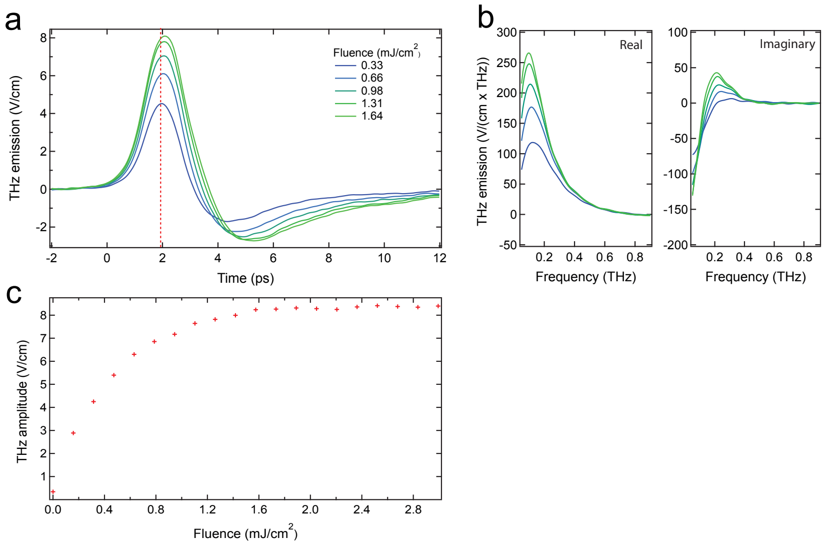

Supplementary Figure 7: Fluence dependence of position 1 in Fig. 2**b**, device A. **a** Emitted field as a function of time, with corresponding real and imaginary part of the emitted field shown in **b** FT results. **c** Fluence dependence of peak amplitude at a time delay 1.933 ps, indicated by the red dashed line in **a**.

In addition, we provide here more position- and fluence-dependent measurements on Device A. The upper and lower panels in the figure below correspond to the same excitation positions as shown in the Fig. 2 (main text), while the middle panel presents the new data at the central area.

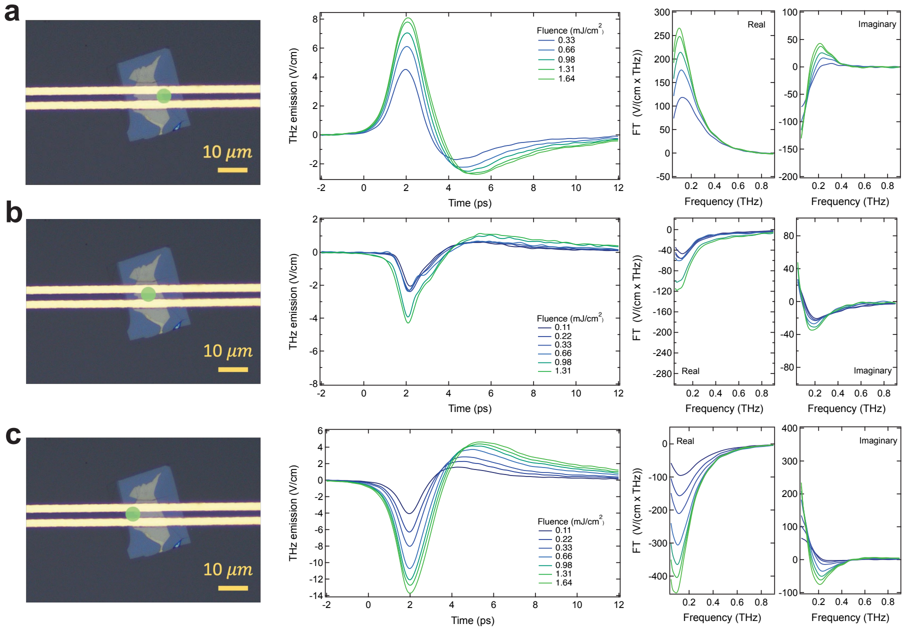

Supplementary Figure 8: **Comparison of photocurrent response for edge and central excitation in Device A.** Upper and lower panels (a, c): excitation positions as in Fig. 2 of the main text; middle panel (b): measurement at the central (non-edge) region between the metal strips, where the emitted signal is much weaker.

When excitation occurs between the metal strips, any possible cavity-related response is strongly damped (See Supplementary Section 8), leaving only the DC photocurrent component detectable. When exciting both between the metal strips and laterally in the middle of the flake, the photocurrent signal under similar excitation fluence is significantly suppressed compared to the edge positions. Due to the limited spatial resolution and potential effects of the presence of the striplines (such as forming edge-like boundaries at the metal strip edges), a perfectly zero signal is not observed. Nevertheless, the photocurrent is clearly maximized when the excitation position approaches the edges of the WTe_2 flakes.

Position dependent measurements were additionally measured on the edges of device C, as shown in Supplementary Figure 9. Consistent with observations of Device A, the emitted field is observed to flip in polarity depending on the excitation edge.

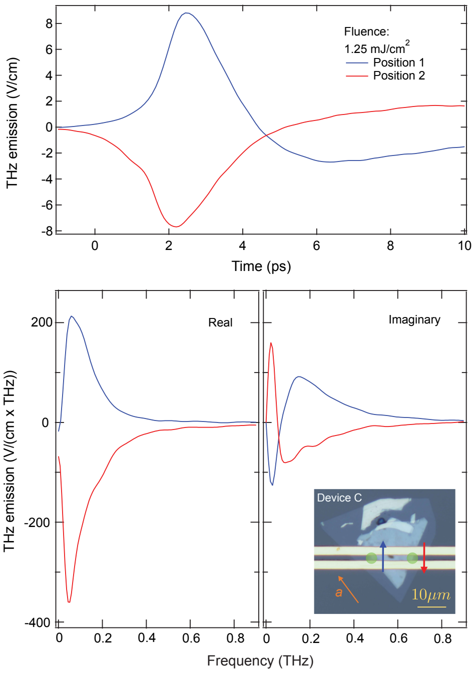

Supplementary Figure 9: Laser excitation in between the co-planar stripline of the left and right edge in Device C, shown in the time domain (top), and corresponding frequency-domain emitted field (bottom).

Supplementary Note 5: Fitting of time domain dynamics

The emitted THz fields are related to the current density in the material through [5],

$$E_{\mathrm{THz}}(t) = -\frac{\partial j_f}{\partial t}., \tag{20}$$

where j_f is the current density of the free carriers.

To identify the processes involved in the emitted signal, the integral of the time traces (corresponding to the photocurrent density j_{ph}) can be fit using the following equation [6]:

$$j_{\mathrm{ph}}(t) = (\mathrm{Erf}((t - t_0)/\tau_1) + 1) \cdot e^{-(t-t_0)/\tau_2} \tag{21}$$

Here, t is the measurement time and t_0 is the time delay of the pump laser, and τ_1 and τ_2, to the timescale of the signal growth and decay respectively. Thus, in this

equation, the first term corresponds to an error function that describes the laser-induced excitation with a time constant τ_1, while the second part accounts for an exponential decay characterized by a time constant τ_2.

Using this formula, the time constants extracted for positions 1 and 2, are $\tau_1 = 1.3$ ps and $\tau_2 = 4.6$ ps, respectively (see Supplementary Figure 10).

These time constants are compared to reference traces obtained when THz pulses are generated and detected by two photoconductive switches using a similar circuit design but with no sample present. In this reference circuit, the time constants were found to be $\tau_1 = 0.7$ ps and $\tau_2 = 1.6$ ps, for a reference circuit built on Device D (and additional reference data from [7]) (Supplementary Figure 10, bottom).

The detected signal can be described by the convolution of the time-varying current density j in the co-planar stripline with the photoconductive switch response function R:

$$j_{\text{detect}} = \int \frac{dj}{dt}(t - t')R(t')dt' \tag{22}$$

In the case of a reference circuit without a sample present, the rise time is determined by the timescale of laser-induced charge generation in the photoconductive switch, followed by the carrier recombination time [8]. In contrast, for WTe_2, photoexcitation is expected to result in a rapid excitation of charges, followed by sub-picosecond carrier thermalization through carrier-carrier scattering, and then a slower carrier-phonon scattering process that lasts for tens of picoseconds [9].

Due to the limitations of the switch response function, the time resolution is not sufficient to detect sub-picosecond processes. Consequently, the rise time ($\tau_1 = 1.3$ ps) can be interpreted as incorporating both the carrier excitation and the rapid decay of carrier-carrier interactions, while the decay time ($\tau_2 = 4.6$ ps) is associated with the carrier-phonon decay process.

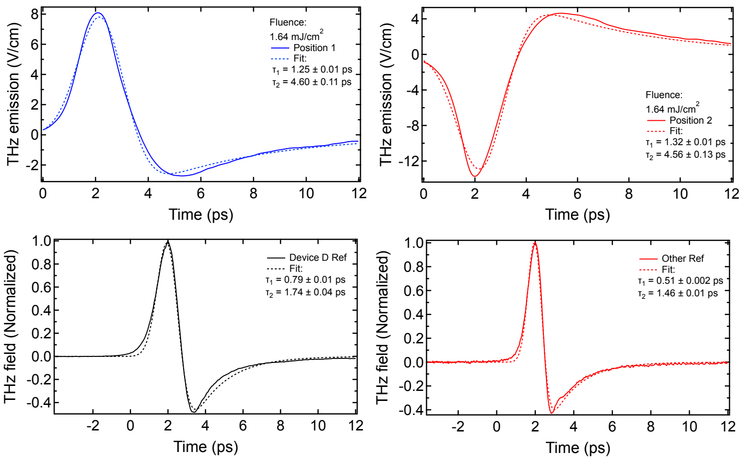

Supplementary Figure 10: **Time domain data fitting** The time domain dynamics and corresponding fit functions are shown for Device A, positions 1 and 2 (top), in comparison to a reference trace from Device D, and an empty circuit from Ref. [7]

Supplementary Note 6: Example of FIR filter for time domain data

The time-domain data with Purcell resonance appears more complex and varies considerably across devices. The reason for this variation is the superposition of different frequency components. To gain a clearer understanding of the time-domain behaviour, one can apply a finite impulse response (FIR) filter. As an example for Device A, shown in Supplementary Figure 11, this analysis reveals a superposition of a single-cycle emission from the DC photocurrent component and a high-frequency oscillation from the Purcell resonance, which agrees well with the insights obtained from the frequency-domain data.

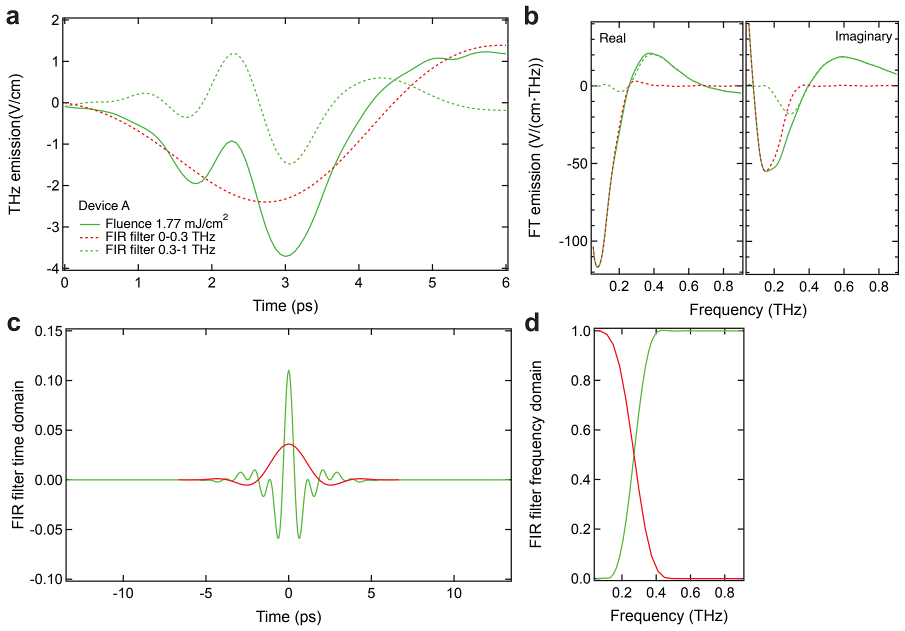

Supplementary Figure 11: **Example of FIR filter applied to highest-fluence measurement of Device A.** (a, b) Time- and frequency-domain traces of Device A at 1.77 mJ/cm^2, corresponding to the highest-fluence trace in Fig. 3 (main text). (c, d) Time and frequency responses of the two FIR filters used; these select frequency components below 0.3 THz and between 0.3–1 THz, respectively.

Supplementary Note 7: Extended data for excitation outside the co-planar stripline

Additional fluence dependent data are shown for Device C (a-b) and Device D (c-d). In contrast to other devices, Device D exhibited Purcell enhancement and directional photocurrent also when photoexcited in between the co-planar stripline in addition to when excited outside, and on an edge. This difference is possibly caused by the thick (64 nm) WTe_2 flake used in Device D.

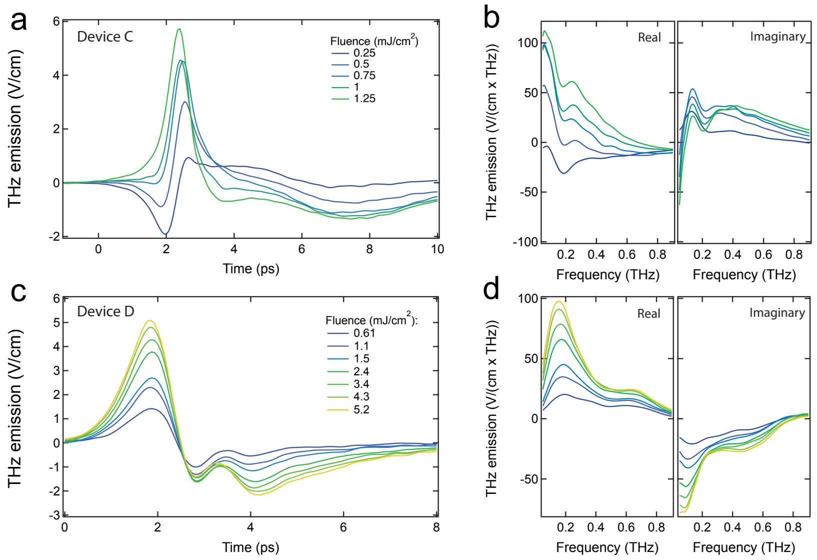

Supplementary Figure 12: Fluence dependence data from Device C and D. **a** and **b** correspond to the time domain and spectral domain emitted fields for Device C when photoexcited at the upper right location outside the co-planar stripline. **c** and **d** display the time domain and spectral domain data for Device D when photoexcited on the left side in between the co-planar stripline. This is the only data that exhibits a Purcell enhanced peak when photoexcited in between the co-planar stripline.

Supplementary Note 8: Cavity theory

To model the Purcell enhancement by the cavity, we introduce the cavity theory discussed in the main text, whose derivation is based on the analytical theory of Ref. [7, 10]. In this work, a current is photoexcited in the sample, which reflects off the edges of the flake and at the discrete changes in the dielectric environment, provided by the metallic co-planar stripline traces. Some wavelengths of excited currents constructively interfere and modify the plasmonic density of states, shifting spectral weight to these frequencies and enhancing emission, whereas other wavelengths destructively interfere, resulting in suppressed current emission. This interference can be quantified by the Purcell factor, $F_{\text{cavity}}(\omega)$, where $\omega = 2\pi f$, which represents the ratio between the modulated current density, $j_{\text{cav},2}(\omega)$, enhanced by self-cavity effects within the co-planar stripline traces and detected via the photoconductive switch, and the intrinsic photocurrent, $j_i(\omega)$, of WTe_2 in the absence of finite-size effects:

$$\left| \frac{j_{\text{cav}}(\omega)}{j_i(\omega)} \right| = F_{\text{cavity}}(\omega) \tag{23}$$

To determine $F_{\text{cavity}}(\omega)$, it is first assumed that the local photocurrent excitation rapidly propagates throughout the device, such that the resulting current can then be described as the sum of the transmitted and reflected currents in each region:

$$j_{\text{cav},a}(x) = r_a e^{-iq_a x} + t_a e^{iq_a x} + j_{i,a}(x) \tag{24}$$

Here, the index a is used to denote the region (0,1,2,3,4), and q the corresponding wavevector in each region.

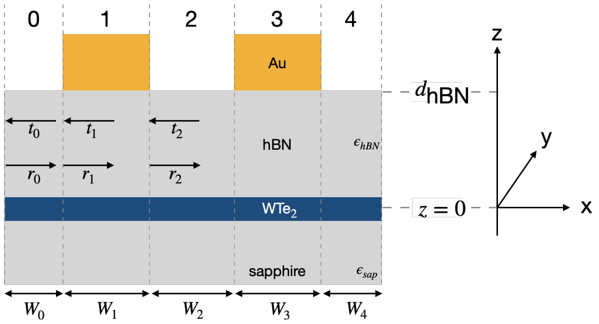

Supplementary Figure 13: Cross section of the heterostructure, with regions labeled. The screening provided by the co-planar stripline traces results in distinct electromagnetic environments. Diagram adapted from [7].

Using Maxwell's equations, one can further derive the potential ($V_a(x)$) in each region.

$$V_a(x) = \frac{1}{\sigma_{2D}}\left(\frac{-i}{q_a} r_a e^{-iq_a x} + \frac{i}{q_a} t_a e^{iq_a x}\right) \tag{25}$$

To find an independent solution for the emitted photocurrent, one next needs to match the current density (j) and potential (V) at each dielectric boundary, combined with the fact that the current density goes to zero at the edges of the flake [11]. This leads to the following boundary conditions [7]:

$$j_a(W_a) = j_{a+1}(0) \qquad \text{for } a = 0, 1, 2, 3 \tag{26}$$

$$V_a(W_a) = V_{a+1}(0) \qquad \text{for } a = 0, 1, 2, 3 \tag{27}$$

$$j_0(0) = 0 \tag{28}$$

$$j_4(W_4) = 0 \qquad , \tag{29}$$

where in each region, the left boundary is defined as $x = 0$ and W corresponds to the width of the region.

The wavevector is determined in each region by the dielectric environment (presence or absence of gold co-planar stripline traces):

$$q_a(\omega) = \begin{cases} q_{\text{screened}} & \text{if } a = 1, 3 \\ q_{\text{unscreened}} & \text{if } a = 0, 2, 4. \end{cases} \tag{30}$$

In regions without the metal traces, the WTe_2 region can sustain an unscreened 2D plasmon. In contrast, in regions 1 and 3, the WTe_2 is screened by the metal traces and hosts screened plasmons. The corresponding wavevectors can be found by inverting the dispersion relation, given by [12, 13]:

$$q_{\text{unscreened}} = \frac{\omega(i\gamma + \omega)(\epsilon_{\text{hBN}} + \epsilon_{\text{sap}})}{\omega_{3D}^2 d_{\text{WTe}_2}}. \tag{31}$$

$$q_{\text{screened}} = \sqrt{\frac{\omega^2 \epsilon_{\text{hBN}} + i\epsilon_{\text{hBN}}\gamma\omega}{\omega_{3D}^2 d_{\text{WTe}_2} d_{\text{hBN}}}}. \tag{32}$$

Where γ is the scattering rate and ω_{3D} the bulk plasmon frequency of WTe_2; ϵ and d are the relative permittivity and thickness of corresponding layers [7, 14]. Meanwhile, we have 2D conductivity:

$$\sigma_{2D} = \frac{\omega_{3D}^2 d}{-i\omega + \gamma} \tag{33}$$

The detection readout using the co-planar stripline is most sensitive to the region in between the stripline traces (corresponding to region 2):

$$j_{\text{cav},2}(x) = r_2 e^{-iq_2 x} + t_2 e^{iq_2 x} + j_{i,2}(x)$$

.

This expression can be further simplified given that, in the unscreened regions $(a = 0, 2, 4)$, $q_a \cdot W_a \ll 1$, $e^{-iqx} \to 1$.

To determine the current density in each region subsequent to photoexcitation, we consider two situations. When we excite in between the co-planar stripline, we assume a uniform current density in region 2, and no initial current density in other regions:

$$j_{i,a} = j_i \delta_{a,2} \tag{34}$$

When the device is photoexcited outside the co-planar stripline, we assume a uniform current density in most of region 0, but goes to zero at the flake edge. The current is expected to exponentially decay as it approaches the edge. The model presented here only depends on the boundary conditions between regions, and so one can ignore the initial spatial distribution within region 0, and instead use:

$$j_i(x) = \begin{cases} j_i & \text{at the boundary of regions 0 \& 1} \\ 0 & \text{at all other boundaries} \end{cases} \tag{35}$$

Using these approximations, the excited current in region 2 can be written as:

$$j_{\text{cav}} = r_2 + t_2 + j_i \delta_{2,b}. \tag{36}$$

Where $\delta_{2,b}$ denotes the excitation location (such that b is either 0 or 2, corresponding to excitation in region 0 or 2).

If one examines the boundary conditions, Eq. 26-29, these consist only of linear combinations of r, t and j_i, so we can define $r \propto \bar{r} j_i$ and $t \propto \bar{t} j_i$.

$$j_{\text{cav}} = (\bar{r}_2 + \bar{t}_2 + \delta_{2,b}) j_i. \tag{37}$$

Thus, the Purcell factor is given by,

$$F_{\text{cavity}}(\omega) = |(\bar{r}_2 + \bar{t}_2 + \delta_{2,b})|. \tag{38}$$

Tunability of the Purcell resonance frequency

To illustrate how the Purcell resonance frequency can be tuned, we present a simplified version of the analytical theory. A precise determination of the resonance frequency requires solving the full boundary equations. However, it can be interpreted qualitatively as a superposition of the resonance frequencies for the screened and unscreened plasmon modes, corresponding to regions with and without the metal strips, respectively.

The unscreened plasmon frequency essentially corresponds to the 2D plasmon frequency of the WTe_2 flake and can be written as:

$$\omega_{\text{unscreened}} = \sqrt{\frac{\omega_{3D}^2 \, q \, d_{\text{WTe}_2}}{\epsilon_{\text{hBN}} + \epsilon_{\text{sapphire}}}} \tag{39}$$

where ω_{3D} is the 3D plasmon frequency along the detection direction (perpendicular to the stripline), d_{WTe_2} is the flake thickness, and ϵ_{hBN} and $\epsilon_{\text{sapphire}}$ are the dielectric constants of the hBN and sapphire layers, respectively. This mode generally lies at a much higher frequency than the upper limit of our detection bandwidth.

The screened plasmon frequency is the dominant term at lower frequencies (within our measurement bandwidth) and can be expressed as:

$$\omega_{\text{screened}} = \sqrt{\frac{\omega_{3D}^2 \, q^2 \, d_{\text{WTe}_2} \, d_{\text{hBN}}}{\epsilon_{\text{hBN}}}} \tag{40}$$

where d_{hBN} is the hBN layer thickness and the wavevector $q \approx \frac{2\pi}{4W_1}$ is determined by W_1, the width of one metal strip. As this term lies within the detectable THz range, it is the primary contributor to the observed Purcell resonance in our experiments.

Equation 40 would correspond exactly to the Purcell resonance in the limit $W_0, W_2, W_4 \to 0$, where only the screened region remains. In this case,

$$\lim_{W_0, W_2, W_4 \to 0} \omega_{\text{Purcell}} = \omega_{\text{screened}}. \tag{41}$$

Under experimental conditions, however, the widths W_0, W_2, and W_4 are finite, and the Purcell resonance is therefore shifted due to hybridisation between screened

and unscreened plasmon modes within the cavity. Nevertheless, ω_{screened} still provides a useful reference for estimating the overall trend of the Purcell resonance frequency:

$$\omega_{\text{Purcell}} \sim \omega_{\text{screened}}. \tag{42}$$

While Eq. 40 as a simplified equation does not yield an exact prediction of the plasmon frequency, it gives a useful sense of how the resonance can be tuned. During fabrication, the most straightforward parameters to control are the thicknesses of the WTe_2 and hBN layers, and the crystal orientation of the WTe_2 flake. For example, in designing Device A, we selected these parameters to position the resonance frequency near the centre of our detection bandwidth (≈ 0.5 THz).

Supplementary Note 9: Parameters for cavity theory

To model the properties of WTe_2, we used for the dielectric constant of insulating layer and substrate $\epsilon_{\text{hBN}} = 3.7$ [15], and $\epsilon_{\text{sapphire}} = 10$.

The 3D plasmon frequency $\omega_{\text{plasmon},3D}$ for modeling each sample was taken as the direction perpendicular to co-planar stripline. According to literature, the bulk plasmon frequency is estimated to be $\omega_{3D,a} = 0.7031 \pm 0.017eV$ and $\omega_{3D,b} = 0.4591 \pm 0.045eV$ along a- or b-axis respectively. [16–18]. To calculate the plasma frequency, the following formula was used:

$$\omega_{3D,\text{esti}} = \sqrt{(\cos\theta \cdot \omega_{3D,a})^2 + (\sin\theta \cdot \omega_{3D,b})^2} \tag{43}$$

Where θ is the angle between the a-axis as shown in Supplementary Section 2 and detection direction (perpendicular to the co-planar stripline).

Supplementary Note 10: Purcell factors for each device

In the experimental setup for Fig. 3 (main text), excitation is outside of stripline, corresponding to $b = 0$ for $\delta_{2,b}$. An example for $F_{\text{cavity}}(\omega) = |(\bar{r}_2 + \bar{t}_2)|$ is Fig. 4**a** (main text), using $0.2\gamma_{\text{fit}}$. Where γ_{fit} is the fitting value of the highest fluence (1.77 mJ/cm^2 for Device A).

Similarly, we plot the Purcell factor for devices B-D as seen in Supplementary Figure 14**b-d**, assuming $0.2\gamma_{\text{fit}}$ got from each device.

The Purcell resonance frequency in each device is influenced by various device parameters. The dominant contribution comes from the screened plasmon frequency (Eq. 40), although in practice it is a superposition of the screened and unscreened plasmon frequencies (Eq. 39), determined by the boundary equations 26–29. Consequently, the width of the metal strip and the gaps between them (W_1, W_2, and W_3 in Supplementary Figure 13) also affect the resonance.

While the basic geometric parameters are already summarised in Supplementary Table 2 and Supplementary Figure 4, Supplementary Table 4 provides a complete list

of parameters used for calculating the Purcell resonance frequency for each device. The values of W_1–W_3 are based on AFM measurements at the excitation position.

The uncertainty in the orientation angle θ is estimated to be 3–6°, depending on the device. The uncertainties in the WTe_2 and hBN thicknesses are approximately 1 nm, while the uncertainties in the stripline widths W_1–W_3 are about 0.2 µm. These uncertainties are significantly larger than the intrinsic measurement accuracy of the corresponding techniques (SHG and AFM). Instead, they primarily reflect variations arising from data collected at different measurement positions across each device.

Supplementary Table 4: Key device parameters: thicknesses, orientation angle (a-axis to detection direction, perpendicular to stripline), and stripline geometry.

Device	θ (deg)	$d_{\mathrm{WTe_2}}$ (nm)	d_{hBN} (nm)	W_1 (µm)	W_2 (µm)	W_3 (µm)
A	22	10.0	26.0	2.8	2.1	3.0
B	46	6.8	8.3	3.1	2.6	3.3
C	36	10.0	16.5	2.8	2.2	2.8
D	26	64.0	25.0	3.4	3.1	2.8

The spectrum for Device B contains two resonances. The lower frequency peak matches that observed in the experiments, whereas the higher-frequency resonance is not observed. We did not expect to see significant enhancement at the higher frequency peak ($\sim$ 0.7 THz) for a few factors, and thus focused our analysis on the lower frequency mode. First, the Purcell enhancement is a resonant process, as the initial directional photocurrent spectrum (j_i) is projected onto the Purcell factor. If there is negligible directional photocurrent resonant to the cavity mode, no emission enhancement will be observed. Second, higher order cavity modes are theoretically found to be overdamped as they are much more sensitive to the boundary conditions of the flake. Finally, the measured field is convolved with the switch response function, which was not independently measured and not included in the theory. For this reason, higher frequencies will be attenuated by the bandwidth of the measurement. Thus, the frequency upturn for Device A,C,D are also not expected to be observed in the experimental data. Future comparisons between the measured spectral lineshapes, and a quantitative analysis of the actual magnitude of the Purcell factor, are left for future work.

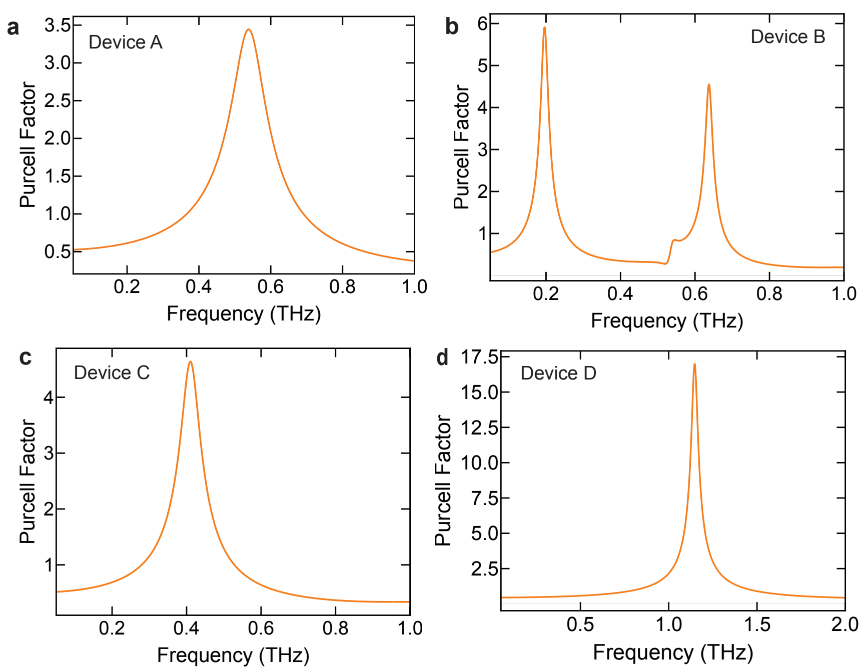

Supplementary Figure 14: **Purcell factor for devices A-D. a** Purcell factor for Device A ($\gamma = 0.2\gamma_{\text{fit}}$), same as Fig. 4**a** (main text). **b** Purcell factor for device B. The lower frequency peak is compared to the experimental data. **c** and **d** show the Purcell enhancement for devices C and D, respectively.

From the experimental data, we observe a consistent redshift of the resonance peak as fluence increases (see Figs. 3c and 3f, main text). We fit this behaviour using a damped-harmonic-oscillator model, which yields the undamped resonance frequency (shown as dashed lines in those panels). This undamped frequency corresponds to the ideal case of the plasmonic cavity. In our comparison with theory, we use this undamped frequency rather than the peak frequency at a given fluence.

In the analytical model, the damping is represented by a parameter γ. To illustrate the idealised situation, we calculated the Purcell factor in the limit $\gamma \to 0$, producing a well-defined resonance frequency:

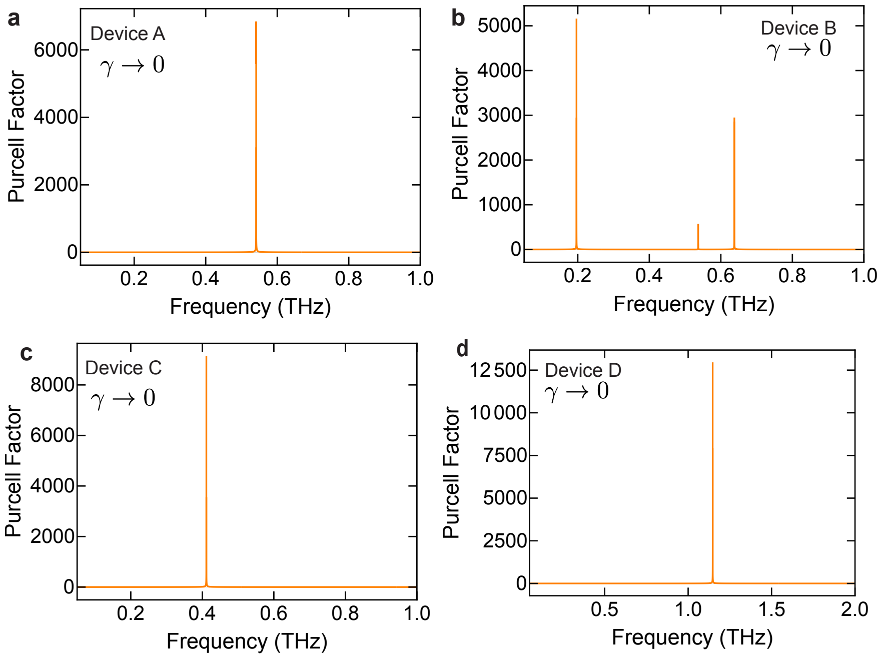

Supplementary Figure 15: Purcell factors in the ideal ($\gamma = 0.0001\gamma_{\mathbf{fit}}$) case for all devices. Represents undamped cavity resonances with high quality factors.

This $\gamma \to 0$ case assumes a cavity with a very high quality factor. Conversely, increasing γ in the model (poorer quality factor) also leads to a redshift of the resonance peak; however, the shift predicted by the simplified model is much smaller than that observed experimentally, which could be attributed to the nonlinear characteristics not captured by the theory.

Therefore, to achieve a meaningful comparison between experiment and theory, Fig. 4b (main text) uses only the ideal-case limit: the undamped resonance frequencies extracted experimentally (dashed lines in Figs. 3c and 3f, lower panels in the main text) are compared with the resonance frequencies calculated in the $\gamma \to 0$ case of the analytical model.

A useful comparison can be made between Supplementary Figures 14 and 15. Supplementary Figure 15 illustrates an idealised undamped case ($\gamma \to 0$; no amplitude decrease in time), in which the resonance peaks reduce to delta functions. When the simulation is adjusted to match experimental conditions, a finite damping ratio is introduced, corresponding to a decay in the photocurrent amplitude over time. In the frequency domain, this temporal decay manifests as a finite linewidth of the resonance (see Fig. 4a in the main text and Supplementary Figure 15), in agreement with our measured spectra. Thus, while formulated in the frequency domain, the model implicitly captures the temporal behaviour of the photocurrent through its linewidth, which encodes the decay rate.

Finally, in the theory the difference in resonance frequencies between the damped and undamped cases is relatively small; thus Fig. 4a (main text) remains a good representation of both the width and the resonance frequency of the Purcell mode.

Supplementary Note 11: Extended model including local heating

The analytical model presented in Supplementary Section 8 successfully describes excitation outside the stripline and accurately predicts the Purcell resonance peak, as shown in Supplementary Section 10. However, it does not fully explain why the Purcell resonance peak is observed only in this geometry and not when excitation occurs between the metal strips.

The qualitative explanation is that direct excitation by the pump laser causes local heating in the illuminated area, which increases the damping rate and drives the cavity into an overdamped regime when excited in the central region between the strips. This heating effect is far less pronounced when the excitation is applied near the edge of the stripline, where part of the cavity is protected (screened) by the metal.

To estimate the possible effect of laser heating on the damping, we note that the optical constants of WTe_2 along the b-axis at 515 nm ($n \approx 3.2$, $k \approx 1.6$ [19]) give a reflectivity $R = \frac{(n-1)^2+k^2}{(n+1)^2+k^2} \approx 0.366$ and absorbed fraction $A_{\mathrm{abs}} \approx 0.634$. The optical penetration depth is only $\delta \approx 5\,\mathrm{nm}$ [20], so the absorbed energy density for a fluence F is $E_{\mathrm{vol}} \approx F A_{\mathrm{abs}}/\delta$. At low temperatures, the volumetric heat capacity follows the Debye law $C_{\mathrm{vol}}(T) = \beta T^3$ with $\beta \approx 2.4 \times 10^{-5}\,\mathrm{J/(cm^3\,K^4)}$ [21, 22], giving the peak temperature after a single pulse as $T \approx \left(T_0^4 + \frac{4FA_{\mathrm{abs}}}{\beta\delta}\right)^{1/4}$. For typical conditions ($T_0 = 20\,\mathrm{K}$, $F = 3\,\mathrm{mJ/cm^2}$) this yields $T/T_0 \approx 8$.

In this material, the resistivity, ρ, scales as $\rho \propto T^2$ at low T [23]. The Drude model assumes that $\gamma \propto \rho$. Thus, one could expect that the linewidth will change as $\gamma/\gamma_0 \approx (T/T_0)^2 \approx 64$. This instantaneous heating thus could lead to an increase in damping on the order of 50, and thus we use in these calculations $\gamma \sim 50\gamma_0$ in the below estimates.

We extend our analytical model to include region-dependent damping coefficients γ_i.

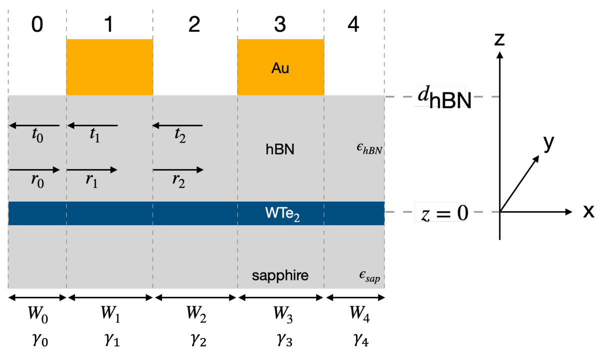

Supplementary Figure 16: **Cross section of the heterostructure with labeled regions.** Extended version of Supplementary Figure 13. In this model, each spatial region is assigned its own damping coefficient γ_i.

For areas not directly illuminated by the laser, we set $\gamma_i \equiv \gamma_{\text{cav}}$, which corresponds to the experimentally extracted cavity damping.

For areas directly subject to laser illumination, we compared two cases: 1. $\gamma_i = \gamma_{\text{cav}}$ (no additional heating effect); 2. $\gamma_i = 50\,\gamma_{\text{cav}}$ (representing a strong heating-induced increase in damping).

This region-dependent scattering rate γ_i modifies both the wavevector $q_a(\omega)$ and the two-dimensional conductivity $\sigma_{2\text{D}}$ in each region, as defined by Eqs. 30, 31, 32, and 33. These two quantities enter the field equations [Eqs. 24 and 25] for each region, and, after applying the updated boundary conditions [Eqs. 26–29], yield new solutions for the Purcell factor under various excitation geometries.

The calculated Purcell factors for Device A in all four cases are shown below:

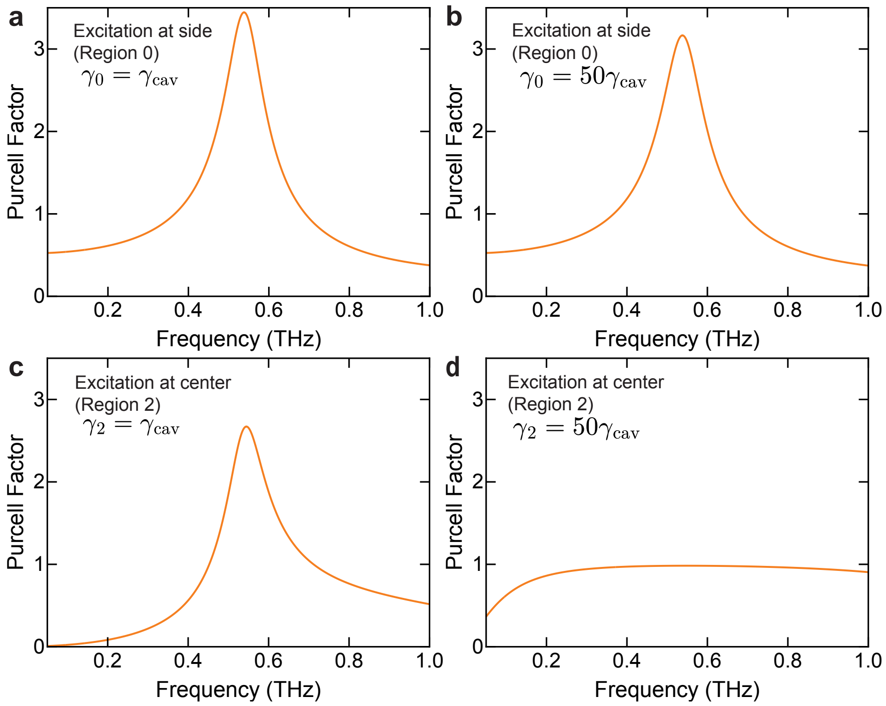

Supplementary Figure 17: Simulated Purcell factors for Device A under various damping conditions. Cases include excitation at region 0 (outside stripline) and region 2 (inside stripline gap), each evaluated with and without increased damping from local heating. The lower cut-off of the x-axis is 50 GHz, corresponding to the lower limit of our experimental bandwidth.

The simulation results show that when the laser excites region 0, the Purcell factor remains essentially unchanged in both low- and high-damping cases. This indicates that our detection is not strongly sensitive to extra damping outside the stripline gap.

In contrast, when the laser excites region 2 (inside the gap), the ideal case without additional damping still produces a Purcell enhancement of similar magnitude to the outside-excitation case. However, when heating increases the damping in region 2 ($\gamma_i = 50\,\gamma_{\mathrm{cav}}$), the Purcell factor is strongly suppressed. Even with a more moderate damping enhancement of $\gamma_i = 8\,\gamma_{\mathrm{cav}}$, the maximum Purcell factor remains only 1.1 when excitation occurs at the center, making it virtually undetectable in the raw data. This supports our physical picture: laser-induced heating in the gap lowers the cavity quality factor enough to push the system into an overdamped regime, eliminating the Purcell resonance peak. In this geometry, only the DC photocurrent component remains detectable, consistent with our experimental observations.

To better illustrate this mechanism, we provide a schematic timing diagram of the photocurrent generation and cavity interaction:

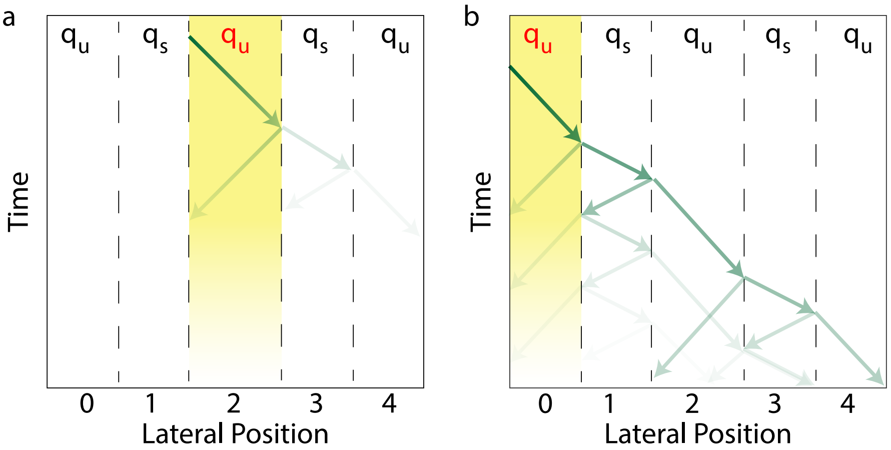

Supplementary Figure 18: **Timing diagram of photocurrent generation.** (a) Excitation between metal stripes: local heating (yellow region) significantly increases damping and pushes the cavity into the overdamped regime, scattering the photocurrent during propagation and suppressing Purcell enhancement (as in Fig. 2 in the main text and Supplementary Figure 7). (b) Excitation outside the stripline: local heating still occurs in the illuminated area but mainly in an off-centre region; a substantial fraction of the photocurrent propagates into the screened region under the metal strip, allowing reflection across the cavity with minimal quality-factor reduction. As a result, Purcell enhancement is preserved (consistent with Fig. 3 in the main text).

This extended modeling quantitatively confirms our qualitative reasoning: the suppression of Purcell enhancement for excitation between strips is due to local heating–induced overdamping, whereas excitation outside the stripline maintains a sufficiently high cavity quality factor for the enhancement to be observed.

Supplementary Note 12: Excitation away from the stripline

Based on the model of local heating induced by the laser (Supplementary Figure 17), moving the pump beam further away from the stripline should yield a similar resonance, as the cavity damping is not significantly affected provided that the excitation occurs outside the metal strips. However, under experimental conditions, several complex factors can influence the final result. Supplementary Figure 19 shows additional position-dependent measurements we performed. The Purcell resonance at a distant position still exists but is less pronounced, due to a combination of effects including the altered excitation edge, dispersion of the photocurrent and complex boundary conditions.

In Supplementary Figure 19, top row corresponds to emission from a position similar to Fig. 3a, while the bottom dataset corresponds to excitation farther from

the stripline. As shown in the fluence dependence in both the time and frequency domains, the photocurrent component decreases and the resonance peak becomes less pronounced as the excitation is moved farther away.

The reduction is hypothesized to occur due to two reasons.

Firstly, when the pump beam is moved far enough to produce a significant difference, the excitation position is ultimately located at a different crystal edge compared to the cases in Fig. 2 or Fig. 3 of the main text. In addition, the longer distance to the cavity introduces greater dispersion of the photocurrent. Taken together, these factors alter the photocurrent lineshape in Device A relative to that shown in Fig. 2 of the main text.

Secondly, excitation at edges far from the cavity structure means that the photocurrent experiences more nonuniform boundary conditions due to the irregular shape of the WTe_2 flake. We deliberately avoided this in our primary measurements by exciting closer to the stripline. Far-edge excitation causes more complex reflections and losses before the resonance builds up, thereby decreasing the formation and detectability of the Purcell peak.

This observation disfavors performing excitation far from the stripline in our experiments. In future work, these effects could be investigated after cutting or etching WTe_2 into well-defined shapes or squares, which would ensure excitation along the same edge while reducing sensitivity to irregular edge-scattering mechanisms.

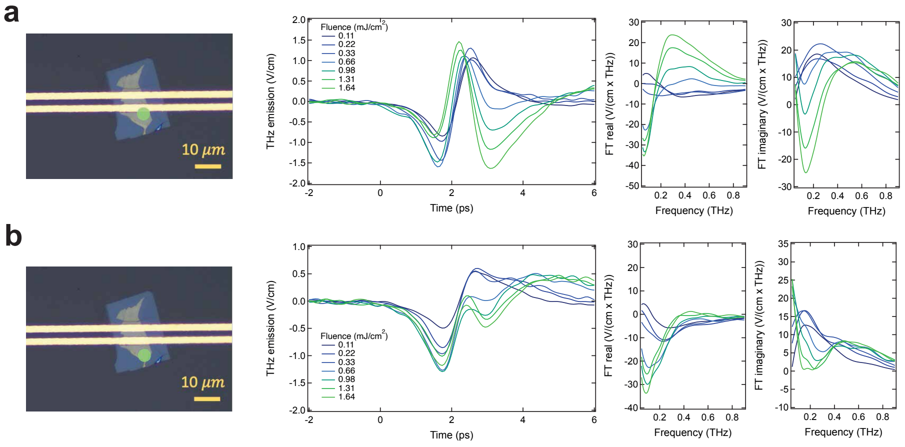

Supplementary Figure 19: **Extra position-dependent measurements for Device A.** Top panels (a): excitation near the cavity edge (similar to Fig. 3a). Bottom panels (b): excitation farther away from the cavity structure, showing reduced photocurrent and disappearance of the resonance peak.

Supplementary Note 13: Field generation efficiency

To compare the efficiency of THz emission of WTe_2 in Fig. 3 (main text), we measured the transmission of a THz pulse launched using a 180 nm evaporated αSi photoconductive switch on Device A, with time-domain traces shown in Supplementary Figure 20**a**. The peak amplitude of THz emission with a laser fluence of 24.6 mJ/cm^2 and bias voltage 10 V is 387.5 V/cm. Second, we measured the photocurrent across the generator switch as a function of bias voltage at a range of laser fluences shown in Supplementary Figure 20**b**. From this data, we can extract a relationship between the laser fluence and expected emitted THz field, with a fit shown in Supplementary Figure 20**d**.

We measured the emitted field at 3.07 ps on WTe_2 Device A as a function of laser fluence. This peak in the time domain is used to approximate the efficiency of the finite frequency peak. At a laser fluence of 2 mJ/cm^2, THz emission reaches a peak amplitude 5 V/cm. Using the calibration shown in Supplementary Figure 20**d**, we can extrapolate to the expected emitted field for silicon with this laser intensity, corresponding to peak of THz emission 70.5 V/cm. As the silicon photoconductive switch is 180 nm thick, this corresponds to an efficiency of 0.39 V/(cm $\cdot$ nm) with 10 V bias voltage. In comparison, WTe_2, which has no voltage bias applied and is 10 nm thick, corresponds to an efficiency 0.5 V/(cm $\cdot$ nm).

We can additionally compare these efficiencies to the recently proposed vdW THz emitter, $NbOI_2$ and established THz emitter ZnTe [24]. With 2 mJ/cm^2, the efficiency of ZnTe is 3.5×10^{-3} V/(cm $\cdot$ nm), and $NbOI_2$, 14×10^{-3} V/(cm $\cdot$ nm), making WTe_2, the most efficient per thickness.

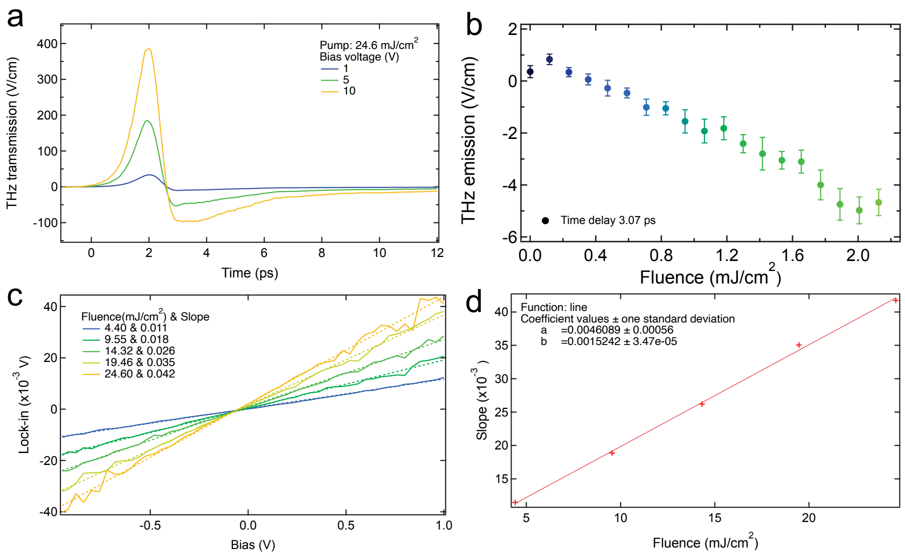

Supplementary Figure 20: **Material efficiency calculation a** Transmission measurement in Device A for different bias voltages on generation switch. **b** Emitted THz field as a function of laser fluence for Device A, measured at 3.07 ps. **c** Switch calibration with different laser fluences for the generation switch. **d** Linear fitting of switch response slope with different laser fluence.

Supplementary References

[1] Wang, Q. *et al.* Robust edge photocurrent response on layered type ii weyl semimetal WTe_2. *Nature communications* **10**, 5736 (2019).

[2] Wang, Y.-X. *et al.* Visualization of bulk and edge photocurrent flow in anisotropic weyl semimetals. *Nature Physics* **19**, 507–514 (2023).

[3] Tiwari, A. *et al.* Giant c-axis nonlinear anomalous hall effect in td-mote2 and wte2. *Nature communications* **12**, 2049 (2021).

[4] McIver, J. W. *et al.* Light-induced anomalous hall effect in graphene. *Nature physics* **16**, 38–41 (2020).

[5] Pettine, J. *et al.* Ultrafast terahertz emission from emerging symmetry-broken materials. *Light: Science & Applications* **12**, 133 (2023).

[6] Duvillaret, L., Garet, F., Roux, J.-F. & Coutaz, J.-L. Analytical modeling and optimization of terahertz time-domain spectroscopy experiments, using photoswitches as antennas. *IEEE Journal of Selected Topics in Quantum Electronics* **7**, 615–623 (2001).

[7] Kipp, G. *et al.* Cavity electrodynamics of van der waals heterostructures. *Nature Physics* 1–8 (2025).

[8] Kumar, M. *The development of on-Chip THz time-domain spectroscopy.* Ph.D. thesis, University of Leeds (2016).

[9] Verma, S. *et al.* A room-temperature ultrafast carrier dynamical study and thickness-dependent investigation of WTe_2 thin films on a flexible pet substrate. *Physica Scripta* **99**, 105985 (2024).

[10] Michael, M. H. *et al.* Resolving self-cavity effects in two-dimensional quantum materials. *Preprint at https://doi.org/10.48550/arXiv.2505.12799* (2025).

[11] Svintsov, D. A. & Alymov, G. V. Refraction laws for two-dimensional plasmons. *Physical Review B* **108**, L121410 (2023).

[12] Yoon, H., Yeung, K. Y., Kim, P. & Ham, D. Plasmonics with two-dimensional conductors. *Philosophical Transactions of the Royal Society A:Mathematical, Physical and Engineering Sciences* **372**, 20130104 (2014).

[13] Economou, E. N. Surface Plasmons in Thin Films. *Phys. Rev.* **182**, 539–554 (1969).

[14] Graef, H. *et al.* Ultra-long wavelength dirac plasmons in graphene capacitors. *Journal of Physics: Materials* **1**, 01LT02 (2018).

[15] Laturia, A., Van de Put, M. L. & Vandenberghe, W. G. Dielectric properties of hexagonal boron nitride and transition metal dichalcogenides: from monolayer to bulk. *npj 2D Materials and Applications* **2**, 6 (2018).

[16] Frenzel, A. J. *et al.* Anisotropic electrodynamics of type-II weyl semimetal candidate WTe_2. *Physical Review B* **95**, 245140 (2017).

[17] Popescu, A., Pertsova, A., Balatsky, A. V. & Woods, L. M. Optical response of $MoTe_2$ and WTe_2 weyl semimetals: Distinguishing between bulk and surface contributions. *Advanced Theory and Simulations* **3**, 1900247 (2020).

[18] Zhu, Z. *et al.* Quantum oscillations, thermoelectric coefficients, and the fermi surface of semimetallic WTe_2. *Physical review letters* **114**, 176601 (2015).

[19] Munkhbat, B., Wróbel, P., Antosiewicz, T. J. & Shegai, T. O. Optical constants of several multilayer transition metal dichalcogenides measured by spectroscopic ellipsometry in the 300–1700 nm range: high index, anisotropy, and hyperbolicity. *ACS photonics* **9**, 2398–2407 (2022).

[20] Buchkov, K. *et al.* Anisotropic optical response of wte2 single crystals studied by ellipsometric analysis. *Nanomaterials* **11**, 2262 (2021).

[21] Callanan, J. E., Hope, G., Weir, R. D. & Westrum Jr, E. F. Thermodynamic properties of tungsten ditelluride (WTe_2) i. the preparation and low temperature heat capacity at temperatures from 6 k to 326 k. *The Journal of Chemical Thermodynamics* **24**, 627–638 (1992).

[22] Laboratory, Q. M. O. Tungsten ditelluride (wte_2) optical properties database (2024). URL https://quantumlab.uark.edu/wte2/. Accessed: 2024-07-06.

[23] Perevalova, A. *et al.* Electronic transport in a topological semimetal wte2 single crystal. *Preprint at https://doi.org/10.48550/arXiv.2302.00297* (2023).

[24] Handa, T. *et al.* Terahertz emission from giant optical rectification in a van der waals material. *Nature Materials* 1–6 (2025).
